# Purification and characterization of a novel cold adapted fungal glucoamylase

**DOI:** 10.1186/s12934-017-0693-x

**Published:** 2017-05-02

**Authors:** Mario Carrasco, Jennifer Alcaíno, Víctor Cifuentes, Marcelo Baeza

**Affiliations:** Departamento de Ciencias Ecológicas, Facultad de Ciencias, Universidad de Chile, Las Palmeras 342, Casilla 653, Santiago, Chile

**Keywords:** Fungal amylase, Cold-adapted amylase, *Tetracladium sp.*, Antarctic fungi

## Abstract

**Background:**

Amylases are used in various industrial processes and a key requirement for the efficiency of these processes is the use of enzymes with high catalytic activity at ambient temperature. Unfortunately, most amylases isolated from bacteria and filamentous fungi have optimal activity above 45 °C and low pH. For example, the most commonly used industrial glucoamylases, a type of amylase that degrades starch to glucose, are produced by *Aspergillus* strains displaying optimal activities at 45–60 °C. Thus, isolating new amylases with optimal activity at ambient temperature is essential for improving industrial processes. In this report, a glucoamylase secreted by the cold-adapted yeast *Tetracladium* sp. was isolated and biochemically characterized.

**Results:**

The effects of physicochemical parameters on enzyme activity were analyzed, and pH and temperature were found to be key factors modulating the glucoamylase activity. The optimal conditions for enzyme activity were 30 °C and pH 6.0, and the *K*_m_ and *k*_cat_ using soluble starch as substrate were 4.5 g/L and 45 min^−1^, respectively. Possible amylase or glucoamylase encoding genes were identified, and their transcript levels using glucose or soluble starch as the sole carbon source were analyzed. Transcription levels were highest in medium supplemented with soluble starch for the potential glucoamylase encoding gene. Comparison of the structural model of the identified *Tetracladium* sp. glucoamylase with the solved structure of the *Hypocrea jecorina* glucoamylase revealed unique structural features that may explain the thermal lability of the glucoamylase from *Tetracladium* sp.

**Conclusion:**

The glucoamylase secreted by *Tetracladium* sp. is a novel cold-adapted enzyme and its properties should render this enzyme suitable for use in industrial processes that require cold-active amylases, such as biofuel production.

**Electronic supplementary material:**

The online version of this article (doi:10.1186/s12934-017-0693-x) contains supplementary material, which is available to authorized users.

## Background

A large proportion of the earth’s biosphere is constantly below 5 °C and these cold environments are inhabited by cold-adapted microorganisms among other forms of life. Cold-adapted yeasts have attracted the attention of scientists because these yeast species have evolved to adapt to cold climates, and thus have significant potential for applications in diverse fields of industry [1–5]. A very well studied feature of cold-adapted yeasts is the presence of hydrolytic enzymes, which are secreted to aid the uptake of nutrients available in their surrounding environment. These cold-active enzymes have many applications in processes requiring high activity at low or mild temperatures [6–8]. Examples are the cold-active amylases, lipases, proteases, cellulases, pectinases and esterases, which are applied in food, wine, textile and detergent industries [4, 9]. Amylases hydrolyze α-glucosidic bonds in starch and according to their catalytic mechanism they are classified into three main groups: (i) α-amylase, which disrupts α-1,4-glycosidic linkages; (ii) β-amylase, which catalyzes the hydrolysis of the second α-1,4 glycosidic bonds from the non-reducing end of starch; and (iii) glucoamylase (an α-glucosidase), which acts on both, α-1,4 and α-1,6 glycosidic bonds from the non-reducing end of the starch molecule [10–13]. Amylases are used in several industrial processes, including the production of high-fructose corn syrup, as additives in detergent formulations, in wool treatment and to obtain fermentable sugars from starch-rich wastes that are used as a substrate for biofuels production [13, 14]. The efficient microbial production of biofuels from raw starch wastes requires the complete degradation of starch, which is currently accomplished by the addition of α-amylase and glucoamylase during the fermentative process to release glucose as the primary end product [15, 16]. The majority of glucoamylases present in bacteria and fungi have optimal activity above 45 °C and at low pH [16–18]. The glucoamylases used in industrial processes, mainly derived from *Aspergillus* strains, display the highest activity at temperatures between 45 and 60 °C [16]. Currently, there is strong interest in finding amylases with better performance at lower temperatures than commercially available amylases, because these enzymes would circumvent the requirement of heating during the reaction process thereby minimizing costs [19]. Several fungi isolated from soil samples from King George Island in the sub-Antarctic region grew on soluble starch as the sole carbon source and displayed extracellular amylase activity. The highest amylase activity was found in samples obtained from the yeast *Tetracladium* sp., and in preliminary characterizations the molecular weight of the enzyme was found to be ~80 kDa [20, 21].

In this report, an amylase from the cold-adapted yeast *Tetracladium* sp. was purified and biochemically characterized. In addition, the amylase encoding gene was identified and its expression was analyzed through RNA-seq when using soluble starch or glucose as the sole carbon source. A model of the enzyme was constructed, revealing several features that are characteristic in cold-adapted enzymes. The optimal conditions for enzyme activity, thermal stability and kinetic parameters were determined. The obtained results suggest that the characterized glucoamylase secreted by *Tetracladium* sp. is a novel cold-adapted enzyme that may be useful in processes where cold-active amylases are required, such as biofuel production.

## Results

### Enzyme purification and characterization

*Tetracladium* sp. extracellular protein samples were obtained by precipitation with ammonium sulfate at 80% saturation of cell-free supernatants of cultures grown using starch as the sole carbon source. Protein separation was attempted using ion-exchange or gel filtration chromatography, obtaining a suitable protein separation only with the last method (Additional file 1). The amylase activity and the protein profile of each fraction were determined. Two main peaks centered at fractions 42 (peak 1) and 72 (peak 2) were observed, but amylase activity was only detected in peak 1 (Additional file 1A). A single protein band of 84 kDa was observed by SDS-PAGE analysis in fractions displaying amylase activity (Additional file 1A). This protein is glycosylated (Additional file 1B, C) and has a relative molecular weight (rMW) of 80 kDa under non-reducing conditions, as determined by gel filtration chromatography.

### Characterization of enzymatic activity

**Fig. 1:**
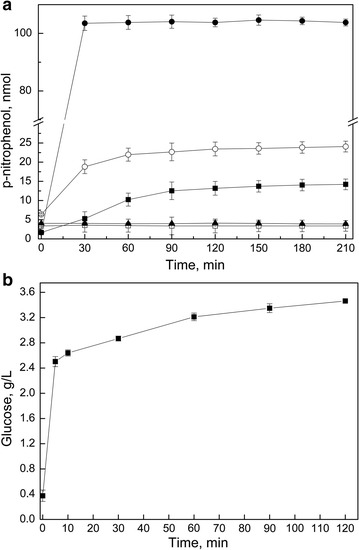
*Tetracladium* sp. amylase substrate specificity. **a** Amylase assays were performed at 30 °C using ethylidene-pNP-G7 (*open symbols*) or 4-nitrophenyl α-d-glucopyranoside (*filled symbols*) as substrates. The release of p-nitrophenol was measured by absorbance at 405 nm, which was quantified using a calibration curve previously constructed with pure p-nitrophenol solutions. Results for the *Tetracladium* sp. amylase are shown as *squares*, commercial α-amylase by *open circles*, commercial alpha-glucosidase by *filled circles* and the negative control (no enzyme) by *triangles*. **b** Assays with the *Tetracladium* sp. amylase were performed using 10 g/L of soluble starch as substrate at pH 6.0 and the release of glucose was followed by the DNS method

To evaluate the specificity of the amylase secreted by *Tetracladium* sp., enzymatic activity assays were performed using either ethylidene-pNP-G7 (E-pNP-G7) or 4-nitrophenyl α-d-glucopyranoside (4-NPGP), which are α-amylase and α-glucosidase substrates, respectively. As shown in Fig. 1a, the enzyme was able to use 4-NPGP as a substrate, but not E-pNP-G7, indicating that the *Tetracladium* sp. enzyme is an α-glucosidase. A characteristic of α-glucosidases is the release of glucose as the end product of starch hydrolysis. To test the possible release of glucose, assays were performed using 5 g/L soluble starch as the substrate and glucose production was quantified during the reaction using the DNS method. As shown in Fig. 1b, the amylase from *Tetracladium* sp. releases glucose from soluble starch, showing that this amylase is an α-glucosidase. Two slopes could be distinguished along the reaction curve; the enzyme had a rapid reaction rate until 10 min reaching 2.5 g/L of glucose and then a slower reaction rate after 10 min (Fig. 1b).

A two-level Plackett–Burman design was applied to determine the influence of temperature, pH, Ca^2+^, Mg^2+^ and soluble starch concentration on enzyme activity. The reaction was followed by measuring the release of glucose in each trial (eight in total) and the reaction velocities were calculated from the slopes of each curve. The effect of each variable was calculated, and it was found that temperature and pH were the two principal factors that influenced strongly the α-glucosidase activity, whereas the ranges tested of soluble starch concentration, and Ca^2+^ and Mg^2+^ levels, affected the enzyme activity to a lesser extent (Additional file 2).

**Fig. 2:**
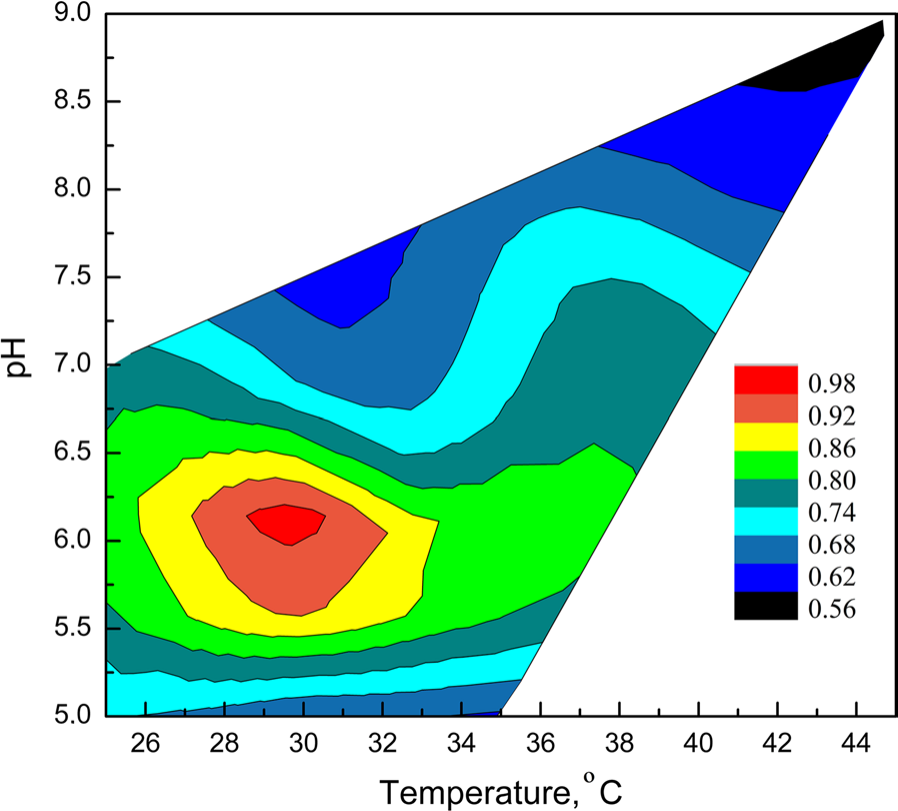
Effect of pH and temperature on the *Tetracladium* sp. glucoamylase activity. Enzymatic reactions were conducted by varying the pH and temperature from the central composite designs. The response surface plot was made following the reaction by the DNS method to determine the glucose released from starch. The glucose concentration (g/L) is represented by the *color scale*

**Fig. 3:**
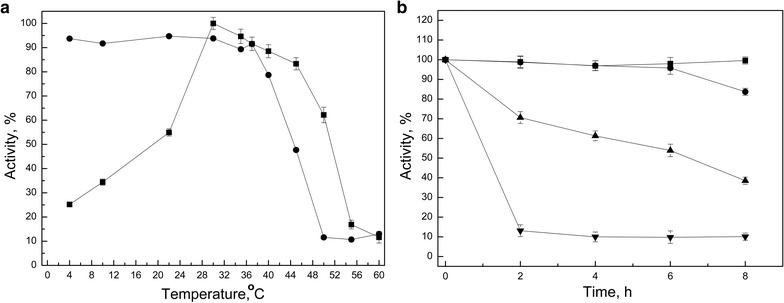
Effect of temperature on the activity and stability of the *Tetracladium* sp. glucoamylase activity. **a** Enzyme activity was assayed following incubation for 1 h at each temperature (*squares*). For thermal stability experiments (*circles*), samples were incubated at the indicated temperatures for 1 h and the remaining activity was determined at 30 °C and pH 6.0. **b** Samples were incubated at 22 (*squares*), 30 (*circles*), 40 (*up triangles*) and 50 °C (*inverted triangles*). The remaining enzyme activity was determined as in **a**

The optimal pH and temperature for α-glucosidase activity on soluble starch were determined using a central composite design of two levels, and it was found that the highest α-glucosidase activity was reached at pH 6.0 and 30 °C (Fig. 2). The enzyme activity and stability were evaluated at temperatures from 4 to 60 °C using soluble starch as the substrate. The highest activity was observed between 30 and 45 °C. At lower and higher temperatures, the enzyme activity decreased significantly, with a more pronounced activity decrease at temperatures above 45 °C (Fig. 3a). At 22 and 4 °C, the enzyme retained 55 and 25% of its maximal activity, respectively.

**Fig. 4:**
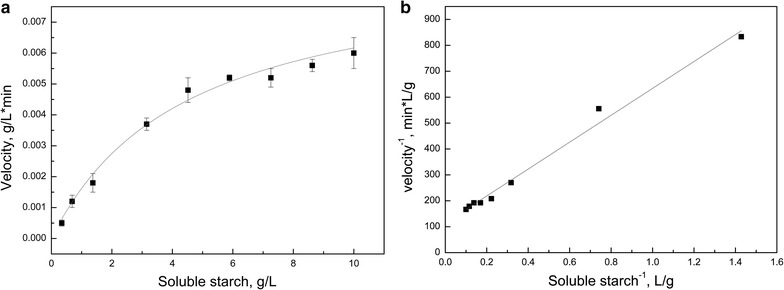
Steady-state kinetic experiments. **a** Enzymatic reactions were carried out by incubating 0.04 µg of the purified *Tetracladium* sp. glucoamylase with different soluble starch concentrations. The reaction rates (*slopes*) of the enzyme reactions were determined at different times and the slope values were plotted against the soluble starch concentration. **b** *Double*-*reciprocal plot* indicating the linear relationship between the reciprocal of the velocity and the reciprocal of the soluble starch concentration

The thermal stability of the enzyme was evaluated by incubating the protein sample for 1 h at different temperatures, and subsequently the enzyme activity was assayed under optimal conditions. This test revealed that the enzyme was stable at temperatures from 4 to 37 °C (Fig. 3a). However, the stability decreased at temperatures above 40 °C, maintaining only 10% of its optimal activity after incubation for 1 h at 50 °C. Figure 3b shows the enzymatic activity after 2–8 h of incubation at different temperatures. The enzyme maintained its activity after incubation at 22 and 30 °C even after 6–8 h of incubation. When incubated at 40 °C, the enzyme activity displayed a linear decrease as a function of incubation period with 70 and 40% of its optimal activity after 2 and 8 h incubation, respectively. The activity loss was very pronounced when the protein sample was incubated at 50 °C for different periods, maintaining only 10% of the maximal activity after only 2 h of incubation at this temperature. Steady-state experiments were used to determine the kinetics of *Tetracladium* sp. α-glucosidase using soluble starch as substrate. Reaction velocities at different soluble starch concentrations and the double-reciprocal plot are shown in Fig. 4. The enzyme displayed Michaelis–Menten kinetics, and the calculated *K*_m_, *k*_cat_ and *k*_cat_/*K*_m_ values were 4.5 g/L, 45 min^−1^ and 10 g/L min, respectively.

### Identification and characterization of the α-glucosidase encoding gene

**Fig. 5:**
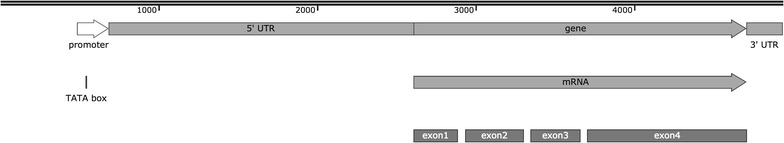
*Tetracladium* sp. glucoamylase gene structure. The mature mRNA has an ORF of 1965 nt that encodes a protein 655 amino acids in length. The identified promoter (200 bp) is shown, which has a TATA-box located at the −40 position from the start site of transcription. The mRNA has a 5′ UTR and a 3′ UTR region of 1928 and 196 bp, respectively. The predicted transcription factors binding sites (SWI6, SAP1 and GCN4) are indicated

The available genomic and transcriptomic data of *Tetracladium* sp. taken from our research efforts were used for the identification and characterization of the gene encoding the α-glucosidase. Bioinformatic tools and the NCBI databases were used to predict and annotate the genes and Open Reading Frames (ORFs) of *Tetracladium* sp. Two putative encoding α-amylase genes and one glucoamylase encoding gene were identified. The expression patterns of these putative genes were evaluated by analysis of the transcriptomes obtained from *Tetracladium* sp. when grown in medium supplemented either with glucose or soluble starch as the sole carbon source. The Fragments Per Kilobase Of Exon Per Million Fragments Mapped (FPKM) values were calculated for the three putative genes and are presented in Table 1. Higher expression levels were found for the putative glucoamylase encoding gene (1.g909.t1), with expression levels similar when either carbon source was used. The two putative α-amylase genes showed expression levels that were approximately tenfold lower than the putative glucoamylase gene. The translated sequences of these three putative genes were compared with peptide mass fingerprint results obtained previously [20]. None of the peptides mapped to putative α-amylase genes, whereas 20 peptides matched the translated sequence of the 1.g909.t1 gene (eight with 100% identity). Twelve of the 20 peptides gave BLAST hits to a fungal glucoamylase precursor deposited in the NCBI protein database. The 1.g909.t1 gene (2.1 kb, sequence in Additional file 3) includes four exons (Fig. 5) with a 1965 nt ORF, which encodes for a protein that is 655 amino acids in length (henceforth called AmyT1). The promoter region was predicted (Fig. 5), which yields a large 5′ UTR region.

**Table 1:** Expression of putative amylase genes of *Tetracladium* sp. in medium supplemented with soluble starch or glucose

Putative gene	Amylase type	FPMK^a^	
		Glucose	Soluble starch
1.g909.t1	Glucoamylase	1545	1722
0.g2254.t1	Alpha amylase	122	184
2.g884.t1	Alpha amylase	76	7

^a^Fragments Per Kilobase Of Exon Per Million Fragments Mapped

**Fig. 6:**
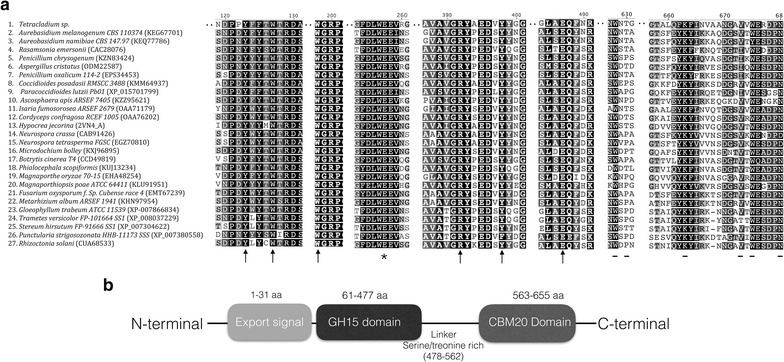
Fungal glucoamylases alignment. **a** Amino acid sequence alignment was performed using fungal glucoamylases deposited in the NCBI database and access numbers are indicated in *parenthesis*. Amino acids involved in substrate binding in the active site, catalysis and in starch binding in the CBM20 domain are indicated by *black arrows*, *asterisks* and the −*symbol*, respectively. **b** Schematic representation of the predicted glucoamylase structure. The export peptide, GH15 domain, CBM20 and linker sequence, are shown

Phylogenetic analysis based on the encoding nucleotide sequence revealed that the *Tetracladium* sp. AmyT1 groups with sequences deposited as hypothetical Coding Sequences (CDS) from *Sclerotinia sclerotiorum* and *Botritys cinerea*, and glycosyl hydrolase CDS from *Phialocephala* and *Trichoderma* species. When the analysis was performed using the deduced protein sequence of AmyT1, the amylase grouped with glucoamylases and hypothetical proteins from *Verticilium* and *Pestalotiopsis* species (Additional file 4). Alignment of the amino acid sequence of AmyT1 and glucoamylases from other fungi showed the presence of conserved residues that are implicated in catalysis (Y47, W51, W120, E179, R309, Y315 and E404) and starch binding (W525, T527, K560, V570, W572 and N577) (Fig. 6). Protein domain analysis predicted that AmyT1 has a 35 amino acid N-terminal export signal, an N-terminal catalytic domain of 407 amino acids, a C-terminal starch binding domain of 108 amino acids and a serine/threonine linker of 47 amino acids between the two domains (Fig. 6b).

## Discussion

According to the biochemical and molecular data presented herein, the glucoamylase Amy1T produced by *Tetracladium* sp. is a novel cold-adapted amylase. The predicted molecular weight based on the ORF is 66 kDa, which is lower than the determined rMW value of 80 kDa observed through SDS-PAGE. This difference is probably due to post-translational glycosylation, which is in accord with previous studies showing that microbial amylases undergo this post-translational modification [22–26]. The optimal pH for activity of the glucoamylase from *Tetracladium* sp. was 6.0, which is similar to the optimum pH value of other microbial glucoamylases [16]. However, its optimal temperature for activity was 30 °C, which is lower than other glucoamylases (i.e., between 40 and 70 °C). Furthermore, the *K*_m_ of the *Tetracladium* sp. glucoamylase towards soluble starch was 4.5 g/L, whereas reported *K*_m_ values for microbial glucoamylases are <1.0 g/L [16]. Generally, cold-adapted or cold-active enzymes have higher *K*_m_ values than their mesophilic or thermophilic counterparts, which is in accordance with our results.

**Fig. 7:**
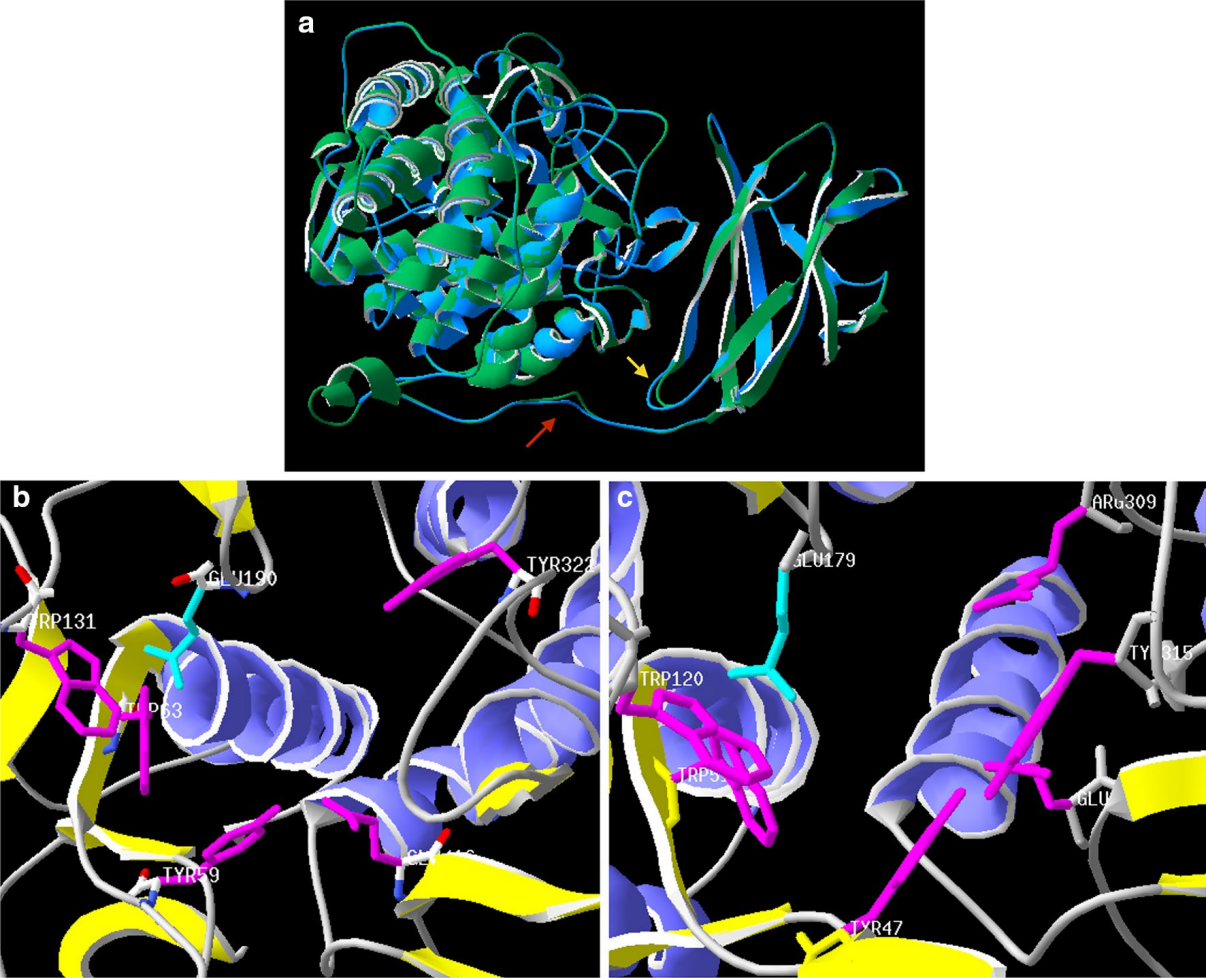
Structural comparison between glucoamylases from *H. jecorina* (HjGa) and *Tetracladium* sp. (AmyT1). **a** Superimposition of the predicted glucoamylase model of AmyT1 (*green*) and the structure of the glucoamylase from HjGa (*blue*). The linker region (*red arrow*) and variable loop (*yellow arrow*) are indicated. Residues involved in substrate recognition and in the catalytic site are shown in *magenta* and *cyan*, respectively, for AmyT1 (**b**) and HjGa (**c**). The calculated distances in Amy1T between residues Y47 and R309, W51 and Y315, W120 and Y315, and E190 and Y315 are 8.5, 9.6, 8.7 and 10.2 Å, respectively. For HjGa, the calculated distances between the equivalent residues Y47 and R309, W51 and Y315, W120 and Y315, and E190 and Y315 were 8.5, 8.6, 8.3 and 10.1 Å, respectively

At the structural level, cold-adapted enzymes are usually more flexible than their thermostable counterparts [27]. The structures of glucoamylases of other fungi such as *Aspergillus awamorii* and *Hypocrea jecorina* (now *Trichoderma reesei*) have been reported [28, 29]. The structure of an enzyme produced by *H. jecorina*, HjGa, was the first crystal structure to include both the catalytic and starch binding domains. This protein shares 69% identity with Amy1T from *Tetracladium* sp., having a 90% coverage. Amy1T was modeled using the crystallographic structure of the glucoamylase from *H. jecorina* (2VN7.1.A) as the template. The superimposition of both structures (Fig. 7a) shows high similarity for both the starch binding domain (SBD) and the catalytic domain (CD). However, differences between the structures were observed mainly for the variable loop of HjGa, which undergoes a large conformational change in the presence of the substrate [29], which is not observed in the Amy1T model. The linker region in the AmyT1 model is 10 residues longer than HjGa and has a lower proline (14.9 vs. 24.3%) and tyrosine (2.1 vs. 5.4%) content. Proline residues have a rigid side chain that confers low flexibility to proteins, whereas tyrosine residues have an aromatic ring that can stabilize protein structures through hydrophobic interactions. These amino acid content differences may explain the more flexible linker of Amy1T when compared with that of HjGa. Generally, cold-adapted enzymes have larger active sites than their mesophilic counterparts [30]; however, no significant differences in the distances between the side chains of the amino acids involved in catalysis between the Amy1T model (Fig. 7b) and HjGa (Fig. 7c) were observed.

As mentioned above, the optimal temperature for activity of glucoamylase Amy1T was 30 °C. Therefore, this enzyme would be considered as a cold-adapted enzyme, but not as a cold-active enzyme. This is supported by the observed thermal stability of the enzyme, which was highly stable at temperatures between 4 and 37 °C, but activity was rapidly lost at temperatures over 40 °C. The stability and optimal temperature of activity of HjGa are between 45 and 65 °C. Numerous interactions between the SBD and CD are important for enzyme activity and stability of HjGa [29], including electrostatic and hydrogen bonding (T589/R27, H560, E652, A80, E157, H600 and D93) and hydrophobic interactions (F76, V594, I604 and V650). The same amino acid types are not present in Amy1T, which may explain the lower thermal stability of Amy1T when compared with that of HjGa.

The activities of fungal glucoamylases are generally affected by calcium [25, 31], with only a few enzymes showing no dependency on this cation for activity [32]. The activity of the glucoamylase from *Tetracladium* sp. showed no calcium dependency. From an industrial application perspective, this is a desirable characteristic because the addition of calcium salts to any process would increase costs and/or give rise to possible secondary effects. In saccharification processes, the pH must be adjusted from 6–6.5 to 4–4.5 prior to the addition of glucoamylase, because most glucoamylases currently used have low activity at pH 6.0–6.5 [33]. Therefore, the amylase from *Tetracladium* sp., that has optimal activity at pH 6.0, represents a good alternative enzyme for these processes. Furthermore, the thermal properties of Amy1T should facilitate process operations at lower temperatures. This feature should save energy and facilitates a simpler enzyme inactivation step by mild heating, and such mild heat denaturation also avoids interfering with downstream steps of the industrial process.

## Conclusions

The glucoamylase secreted by *Tetracladium* sp. is a novel cold-adapted enzyme with optimal activity at pH 6.0 and 30 °C, and has no dependency on Ca^2+^ for its hydrolytic activity. Protein modeling analysis predicted that Amy1T is more flexible than thermostable counterparts, which could explain, at least in part, its higher activity at lower temperatures. The properties of the glucoamylase described in this work should render this enzyme suitable for use in industrial processes that require high starch degrading activity at mild temperatures, such as biofuel production.

## Methods

### Strains and growth conditions

*Tetracladium* sp. was grown in YM medium (yeast extract 0.3%, malt extract 0.3%, peptone 0.5%, pH 7) supplemented with 1% glucose (YM-G) or soluble starch 1% (YM-S). For cultures of 300–500 mL, a decimal volume inoculum at OD_600_ = 12 was used, incubated at 22 °C with 150 rpm orbital agitation. Semisolid media were prepared by the addition of agar at 1.5%, before autoclaving at 121 °C for 20 min. *Tetracladium* sp. is conserved at the Genetic Laboratory Yeast Collection, Faculty of Sciences, Universidad de Chile.

### Extraction and fractioning of extracellular proteins

300 mL cultures of *Tetracladium* sp. at the late exponential phase of growth (OD_600_ = 12) were centrifuged at 7000*g* for 10 min at 4 °C, and the supernatants were filtered through a sterile 0.45-μm pore size polyvinylidene fluoride membrane (Millipore, Billerica, MA, USA). Ammonium sulfate was added to the cell-free supernatants to a final concentration of 80% saturation, incubated on ice for 2 h, and centrifuged at 10,000*g* for 15 min at 4 °C. The pellet was suspended in 2 mL of potassium phosphate buffer (20 mM, pH 7.0 and 150 mM NaCl). For fractioning, ammonium sulfate was added to the supernatants at concentrations from 20 to 80% and proteins were obtained in each fractioning step, as described above. The samples were desalted using a HiTrap desalting column (GE, Schenectady, New York, USA). The protein content of samples was quantified using a BCA assay kit (Thermo Scientific, IL, USA), according to manufacturer’s instructions.

### Amylase purification and glycosylation determination

The total proteins obtained by precipitation with 80% ammonium sulfate were dialyzed through a HiTrap desalting column against a 20 mM sodium phosphate buffer (pH 7.0, 150 mM NaCl). Aliquots of 500 µL protein samples were loaded onto a Superdex 75 10/300 GL column, equilibrated with 20 mM sodium phosphate buffer and a flow rate of 0.2 mL/min attached to an Akta Prime purification system (General Electrics, New York, USA). Fractions of 0.2 mL were collected and analyzed for amylase activity (see below) and protein content by SDS-PAGE. Fractions with amylase activity were pooled and concentrated at 1000*g* and 4 °C using Amicon filters with a 3 kDa molecular weight cut-off.

The rMW of the amylase was determined by comparison against the protein marker bands (PageRuler Plus Prestained Protein Ladder, Thermo Scientific, IL, USA). The calibration curve for the determination of the amylase molecular mass was prepared using a commercial protein standard kit (Gel filtration standard, Bio-Rad, CA, USA).

Glycosylation of the purified enzyme was analyzed by SDS-PAGE stained with the Pierce Glycoprotein Staining Kit (Thermo Scientific, IL, USA).

### Amylase activity determination

The amylase activity in protein extracts was measured as the liberation of reducing sugars from soluble starch by the dinitrosalicylic acid (DNS) method [34]. Briefly, a mixture of 50 µL soluble starch solution at 10 g/L (Sigma-Aldrich Corporation, St Louis, USA) and 50 µL of the protein sample were incubated for 1 h. Then, 100 µLof the DNS (1.6% NaOH, 30% sodium potassium tartrate and 1% 3,5-dinitrosalicylic acid) solution was added, the mixture was incubated for a further 10 min at 100 °C and then for 5 min on ice. The absorbance of the aliquots at 540 nm was measured in 96 well microplates using an Epoch 2 microplate reader (Biotek Instruments Inc., Winooski, VT, USA). The values were normalized by the amount of protein present in each sample. Glucose release from starch was determined using a glucose determination kit (Megazyme, IL, USA).

The specificity of the purified amylase was determined using the chromogenic substrates ethylidene-pNP-G7 (Abnova, Taipei, Taiwan) and 4-Nitrophenyl α-d-glucopyranoside (Sigma-Aldrich Corporation, St Louis, USA) following the supplier instructions. The α-amylase from Abnova and the α-glucosidase from Megazyme were used as positive controls for each specific substrate (glucose/fructose assay kit). The reactions were incubated at 30 °C for different times (0, 5, 10 and 15 min), and at each point the absorbance at 405 nm was measured.

The effect of different factors on the activity of the enzyme was evaluated in a Plackett–Burman design experiment. The parameters assayed were temperature (30, 50 °C), pH (5, 7), concentration of soluble starch (1, 10 g/L), calcium chloride (0, 10 mM) and magnesium chloride (0, 10 mM). Eight different reactions were carried out at different incubation times (between 0 and120 min) and 50 µL samples were taken and assayed by the DNS method, as described above.

To determine the optimum pH and temperature of the reaction catalyzed by the amylase. The reactions were conducted at different temperatures (25, 30, 32.5, 35, 37.5, 40, 42.5 and 45 °C) and pH values (5, 6, 6.5, 7, 7.5, 8, 8.5 and 9). The soluble starch concentration and the incubation time were 10 g/L and 30 min, respectively. Then, 100 µL of the reaction samples were assayed by the DNS method. The concentration of the released reducing sugars at each condition was plotted in a response surface plot.

To determine the kinetic parameters (*K*_m_, *k*_cat_ and *V*_max_), the enzyme concentration was varied such that it gave different reactions rates at different substrate concentrations. The reactions were performed using various soluble starch concentrations (4 to 10 g/L) and different enzyme concentrations (0.8–24.5 µg/mL). In the kinetic assays, the reactions were carried out at the determined optimum pH and temperature (6 and 30 °C) and 0.04 µg glucoamylase. Fifty microliter samples were taken at different time points (0, 30, 60, 90, 120, 150, 180 and 220 min) and assayed by the DNS method. The DNS values obtained in each condition were plotted against the reaction time for each substrate concentration. The slope of the linear phase of the reaction was determined to give the reaction rate. Subsequently, the kinetic parameters were determined using a double-reciprocal plot.

The activity of the glucoamylase at different temperatures was evaluated by incubating 50 µL glucoamylase solutions (6.1 µg/mL) at temperatures from 4 to 60 °C at pH 6 for 1 h. The amylase activity was then determined by the DNS method. The thermal stability of the enzyme was evaluated by incubating enzyme samples at temperatures from 4 to 60 °C for 1 h, prior to the determination of enzyme activity at the optimal conditions. Additionally, kinetic stability was determined by incubating samples at 22–50 °C for 0–8 h using the same procedure.

### Alignment, modeling and bioinformatics analysis

Amino acid sequence alignments were made using the Geneious program v10. The glucoamylases sequences chosen for the amino acid sequence comparison had a minimum of 50% similarity and 50% coverage. The promoter prediction was performed using the FindM tool available in the single search analysis server (http://ccg.vital-it.ch/ssa/findm.php). The glucoamylase model was constructed using the Swiss-model platform [35]. The 2VN4_A PDB entry was chosen for modeling, which is the crystal structure of *Hypocrea jecorina* glucoamylase (HjGa), which has 90% coverage and 69% identity with AmyT1. Distance calculations and models were created using the spdb viewer [36].

## Additional files

**Additional file 1.** Purification and characterization of the amylase enzyme. A, protein samples were loaded onto a Superdex 75 10/300 GL column equilibrated with 20 mM sodium phosphate buffer pH 7.0 and 150 mM NaCl, with a flow rate of 0.2 ml/min. The absorbance values at 280 nm (continuous line) and amylase activity (dotted line) were measured. The SDS PAGE analysis shows the active fractions (lanes 38 to 45). M, protein marker; E, sample from 80% ammonium sulfate precipitation. The identified amylase enzyme is indicated by the arrow. B and C show SDS PAGE gels stained with Coomassie blue reagent or a glycoprotein staining kit, respectively. Lanes 1 and 4, amylase from *Tetracladium* sp.; lanes 2 and 5, horse peroxidase glycoprotein (positive control); and lanes 3 and 6 trypsin inhibitor soybean protein (negative control).

**Additional file 2.** Influence of different parameters on AmyT1 activity.

**Additional file 3.** Sequence of ORF encoding for AmyT1 from *Tetracladium* sp. exons are indicated in grey.

**Additional file 4.** Molecular Phylogenetic analysis by Maximum Likelihood method. A, Based on the encoding nucleotide sequences; B, Based on the translated sequences. All positions with less than 95% site coverage were eliminated, that is, fewer than 5% alignment gaps, missing data, and ambiguous bases were allowed at any position. Evolutionary analyses were conducted using MEGA7. Box, Sequences from *Tetracladium* sp.

## References

[1] Buzzini P, Margesin R. Cold-adapted yeasts: a lesson from the cold and a challenge for the XXI century. In: Cold-adapted Yeasts. Berlin: Springer; 2014. p. 3–22.

[2] Margesin R, Miteva V (2011). Diversity and ecology of psychrophilic microorganisms. Res Microbiol.

[3] D’Amico S, Claverie P, Collins T, Georlette D, Gratia E, Hoyoux A, Meuwis MA, Feller G, Gerday C (2002). Molecular basis of cold adaptation. Philos Trans R Soc Lond B Biol Sci.

[4] Białkowska A, Turkiewicz M. Miscellaneous cold-active yeast enzymes of industrial importance. In: Cold-adapted yeasts: biodiversity, adaptation strategies and biotechnological significance. Berlin: Springer; 2014 p. 377–395.

[5] Alcaíno J, Cifuentes V, Baeza M (2015). Physiological adaptations of yeasts living in cold environments and their potential applications. World J Microbiol Biotechnol.

[6] Cavicchioli R, Charlton T, Ertan H, Mohd Omar S, Siddiqui KS, Williams TJ (2011). Biotechnological uses of enzymes from psychrophiles. Microb Biotechnol.

[7] Feller G (2013). Psychrophilic enzymes: from folding to function and biotechnology. Scientifica (Cairo)..

[8] Gerday C, Buzzini P, Margesin R (2014). Fundamentals of cold-active enzymes. Cold-adapted yeasts: biodiversity, adaptation strategies and biotechnological significance.

[9] Sarmiento F, Peralta R, Blamey JM (2015). Cold and hot extremozymes: industrial relevance and current trends. Front Bioeng Biotechnol..

[10] Janeček Š, Svensson B, MacGregor EA (2014). α-Amylase: an enzyme specificity found in various families of glycoside hydrolases. Cell Mol Life Sci.

[11] Janeček Š, Ševčík J (1999). The evolution of starch-binding domain. FEBS Lett.

[12] Gupta R, Gigras P, Mohapatra H, Goswami VK, Chauhan B (2003). Microbial α-amylases: a biotechnological perspective. Process Biochem.

[13] Gurung N, Ray S, Bose S, Rai V (2013). A broader view: microbial enzymes and their relevance in industries, medicine, and beyond. Biomed Res Int.

[14] Uthumporn U, Shariffa YN, Karim AA (2012). Hydrolysis of native and heat-treated starches at sub-gelatinization temperature using granular starch hydrolyzing enzyme. Appl Biochem Biotechnol.

[15] van Zyl WH, Bloom M, Viktor MJ (2012). Engineering yeasts for raw starch conversion. Appl Microbiol Biotechnol.

[16] Kumar P, Satyanarayana T (2009). Microbial glucoamylases: characteristics and applications. Crit Rev Biotechnol.

[17] Michelin M, Ruller R, Ward RJ, Moraes LA, Jorge JA, Terenzi HF, Polizeli ML (2008). Purification and biochemical characterization of a thermostable extracellular glucoamylase produced by the thermotolerant fungus *Paecilomyces variotii*. J Ind Microbiol Biotechnol.

[18] Aquino ACMM, Jorge JA, Terenzi HF, Polizeli MLTM (2001). Thermostable glucose-tolerant glucoamylase produced by the thermophilic fungus *Scytalidium thermophilum*. Folia Microbiol.

[19] Yingling B, Li C, Honglin W, Xiwen Y, Zongcheng Y (2011). Multi-objective optimization of bioethanol production during cold enzyme starch hydrolysis in very high gravity cassava mash. Bioresour Technol.

[20] Carrasco M, Villarreal P, Barahona S, Alcaíno J, Cifuentes V, Baeza M (2016). Screening and characterization of amylase and cellulase activities in psychrotolerant yeasts. BMC Microbiol.

[21] Carrasco M, Rozas JM, Barahona S, Alcaíno J, Cifuentes V, Baeza M (2012). Diversity and extracellular enzymatic activities of yeasts isolated from King George Island, the sub-Antarctic region. BMC Microbiol.

[22] de Barros MC, do Nascimento Silva R, Ramada MH, Galdino AS, de Moraes LM, Torres FA, Ulhoa CJ (2009). The influence of N-glycosylation on biochemical properties of Amy1, an alpha-amylase from the yeast *Cryptococcus flavus*. Carbohydr Res.

[23] Eriksen SH, Jensen B, Olsen J (1998). Effect of N-linked glycosylation on secretion, activity, and stability of α-amylase from *Aspergillus oryzae*. Curr Microbiol.

[24] Prieto JA, Bort BR, Martínez J, Randez-Gil F, Sanz P, Buesa C (1995). Purification and characterization of a new α-amylase of intermediate thermal stability from the yeast *Lipomyces kononenkoae*. Biochem Cell Biol.

[25] Vihinen M, Mantsiila P (1989). Microbial amylolytic enzyme. Crit Rev Biochem Mol Biol.

[26] Wanderley KJ, Torres FAG, Moraes LÄMP, Ulhoa CJ (2004). Biochemical characterization of alpha-amylase from the yeast *Cryptococcus flavus*. FEMS Microbiol Lett.

[27] Siddiqui KS, Cavicchioli R (2006). Cold-adapted enzymes. Annu Rev Biochem.

[28] Marín-Navarro J, Polaina J (2011). Glucoamylases: structural and biotechnological aspects. Appl Microbiol Biotechnol.

[29] Bott R, Saldajeno M, Cuevas W, Ward D, Scheffers M, Aehle W, Karkehabadi S, Sandgren M, Hansson H (2008). Three-dimensional structure of an intact glycoside hydrolase family 15 glucoamylase from *Hypocrea jecorina*. Biochemistry.

[30] Tsigos I, Mavromatis K, Tzanodaskalaki M, Pozidis C, Kokkinidis M, Bouriotis V (2001). Engineering the properties of a cold active enzyme through rational redesign of the active site. Eur J Biochem.

[31] Benassi VM, Pasin TM, Facchini FD, Jorge JA, Polizeli MLTM (2014). A novel glucoamylase activated by manganese and calcium produced in submerged fermentation by *Aspergillus phoenicis*. J Basic Microbiol.

[32] Soni SK, Kaur A, Gupta JK (2003). A solid state fermentation based bacterial α-amylase and fungal glucoamylase system and its suitability for the hydrolysis of wheat starch. Process Biochem.

[33] Xu QS, Yan YS, Feng JX (2016). Efficient hydrolysis of raw starch and ethanol fermentation: a novel raw starch-digesting glucoamylase from *Penicillium oxalicum*. Biotechnol Biofuels.

[34] Miller GL (1959). Use of DNS reagent for the measurement of reducing sugar. Anal Chem.

[35] Biasini M, Bienert S, Waterhouse A, Arnold K, Studer G, Schmidt T, Kiefer F, Gallo Cassarino T, Bertoni M, Bordoli L, Schwede T (2014). SWISS-MODEL: modelling protein tertiary and quaternary structure using evolutionary information. Nucleic Acids Res.

[36] Guex N, Peitsch MC (1997). SWISS-MODEL and the Swiss-Pdb Viewer: an environment for comparative protein modeling. Electrophoresis.

