# Supplementary material for: Purification and characterization of a novel cold adapted fungal glucoamylase
Source: Microb Cell Fact. 2017 May 2;16:75. doi: 10.1186/s12934-017-0693-x (PMC5414198; doi:10.1186/s12934-017-0693-x)
Supplement: Supplementary file 1 — Additional file 1. Purification and characterization of the amylase enzyme. A, protein samples were loaded onto a Superdex 75 10/300 GL column equilibrated with 20 mM sodium phosphate buffer pH 7.0 and 150 mM NaCl, with a flow rate of 0.2 ml/min. The absorbance values at 280 nm (continuous line) and amylase activity (dotted line) were measured. The SDS PAGE analysis shows the active fractions (lanes 38 to 45). M, protein marker; E, sample from 80% ammonium sulfate precipitation. The identified amylase enzyme is indicated by the arrow. B and C show SDS PAGE gels stained with Coomassie blue reagent or a glycoprotein staining kit, respectively. Lanes 1 and 4, amylase from Tetracladium sp.; lanes 2 and 5, horse peroxidase glycoprotein (positive control); and lanes 3 and 6 trypsin inhibitor soybean protein (negative control). [file 12934_2017_693_MOESM1_ESM.pdf]

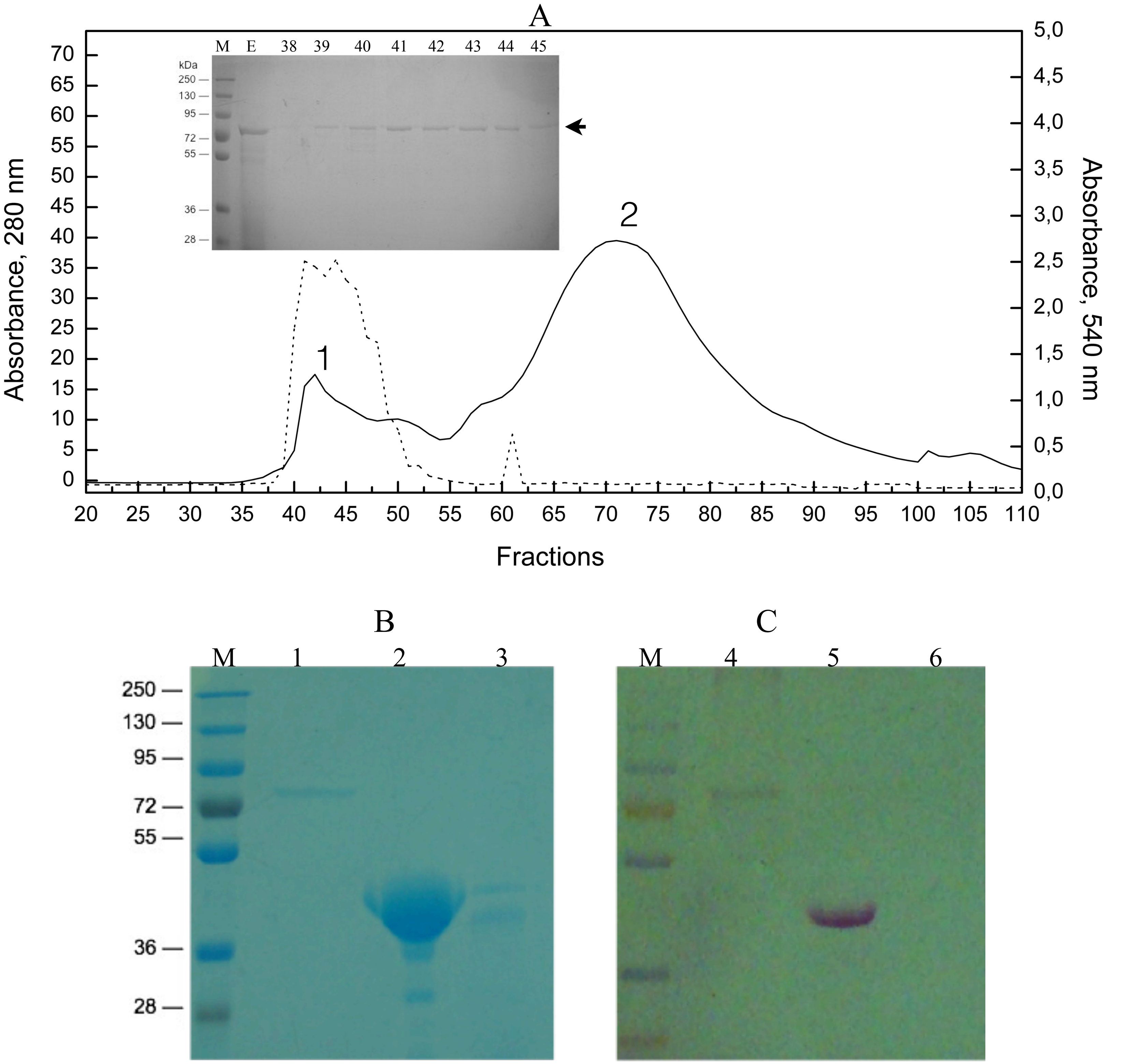

Supplementary Material 1 Purification and characterization of the amylase enzyme**.** A, protein samples were loaded onto a Superdex 75 10/300 GL column equilibrated with 20 mM sodium phosphate buffer pH 7.0 and 150 mM NaCl, with a flow rate of 0.2 ml/min. The absorbance values at 280 nm (continuous line) and amylase activity (dotted line) were measured. The SDS PAGE analysis shows the active fractions (lanes 38 to 45). M, protein marker; E, sample from 80% ammonium sulfate precipitation. The identified amylase enzyme is indicated by the arrow. B and C show SDS PAGE gels stained with Coomassie blue reagent or a glycoprotein staining kit, respectively. Lanes 1 and 4, amylase from *Tetracladium* sp.; lanes 2 and 5, horse peroxidase glycoprotein (positive control); and lanes 3 and 6 trypsin inhibitor soybean protein (negative control).
