# Supplementary material for: Purification and characterization of a novel cold adapted fungal glucoamylase
Source: Microb Cell Fact. 2017 May 2;16:75. doi: 10.1186/s12934-017-0693-x (PMC5414198; doi:10.1186/s12934-017-0693-x)
Supplement: Supplementary file 2 — Additional file 2. Influence of different parameters on AmyT1 activity. [file 12934_2017_693_MOESM2_ESM.pdf]

Supplementary Material 2.

Influence of different parameters on AmyT1 activity.

	High value	Low value	Effect
Temperature (ºC)	50	30	2.8
pH	8	6	1.9
Soluble starch (g/L)	5	0.5	1.0
$CaCl_2$ (mM)	10	0*	0.9
$MgCl_2$ (mM)	10	0*	0.2

A Plackett-Burman design was applied. The effect corresponds to ratio between influence of each parameter and the experimental error. *, no compounds addition.
