# Supplementary material for: Purification and characterization of a novel cold adapted fungal glucoamylase
Source: Microb Cell Fact. 2017 May 2;16:75. doi: 10.1186/s12934-017-0693-x (PMC5414198; doi:10.1186/s12934-017-0693-x)
Supplement: Supplementary file 3 — Additional file 3. Sequence of ORF encoding for AmyT1 from Tetracladium sp. exons are indicated in grey. [file 12934_2017_693_MOESM3_ESM.pdf]

```
atgttctccttcttctctcagcttcagttcctcactctcaagcctctcatctctt 55
tagccatcatggttactttctcctctctcttcctcactgtaggcctcgcatcaca 110
agtcgtcctctcacttccaaatcagaagcgtgatgttgactcttttattgcgaga 165
gagtctcctatagcttacaatggcatactgtgcaacattggcccaagcggagcat 220
gtgtttcgggagctgggtctggtttggtgattgccagtccttcaaggaacgaccc 275
accatgtatgtttctccttgaataccctcatcttataaaactgacggcttagac 330
ttctttacttggactcgtgatgccgcgttggttttcaagtacctcgtggatcgtt 385
ttgtcgagacttacgactcttctcttcaaatccagattcagcgttacatcacctc 440
tcaagcaaaacttcagactgtgatcaatccttccgggggtcttgcaaatggaggt 495
cttggtgaagcaaagtacaatgctgatggtactcaatttactggtgactggggtc 550
gtccacagcgcgatggaccagctctgcgtgctactgcactcattacttatgccaa 605
atggttggtcgcgaatgggtatacgtctacagcacagtctcttgtatggcctgtt 660
atccgcaacgatctctcatacgtgacgcaatactggtaagatgatgccatgacat 715
ttttactatcagctaacagatagtaggaaccagactggctttgatctctgggaag 770
aggttcggggctctagtttcttcactattgcagtctctcaccgtgctcttgttga 825
aggtagtgccctggccgctcagcttgggacatcttgtacctactgcgattcccaa 880
gcccccccaggttttgtgcttcttgcaacgattttggcaaccaaacaatggtggct 935
acattctttcaaacatcaacgtcaacgatggacgttccaagaaggatgcaaacag 990
tatcttgacttcgattcacaactttgaccctaatgtgggatgtgacgccactaca 1045
tatcagccgtaagtactctttccagtttctgatacaattcactaacccttataga 1100
tgctccgatcgtgctctgtcaaatcacaaagccgtcactgattctttccgctcaa 1155
tctatggcatcaactctggaaagggagccggacaagctgtcgctgtaggacgata 1210
tgctgaagatgtctactatggtggcaacccatggttcttggccaaccttgctgct 1265
gcagaacagctgtacgatgcattatacacatggaacaagcagggttctatcactg 1320
tgacttccgtctcccttggcttcttcagggatctcgtatcctctgtcaatgtcgg 1375
cacataccagtcctcaagctcactttataccaccatctacaatgccgtcagggtc 1430
tatgctgatggatatgttgcaatcgttgagcaatacgccgaagcagatggtggtc 1485
ttgccgaacaattcaaccgcaacaatggatcaccactttccgctgctgacctcac 1540
ttggtcatacgctgctttcctcacagcagcagatcgtcgccgagctgctgttcct 1595
tacccatggattgtcccctctgccacgaatgttccaggtcaatgtgttgctacat 1650
ccgcatttggcacatactctgctgcaccgactagcacattccctccaaaccaaac 1705
cccaactggaggagttccaaccacgaccgctcgccccacatcaacgggcacaaga 1760
accacaacatcaggagctccatgtgccactgccacgacagtcgctgtgacattca 1815
atgtccttgccactactcaatttggtcaaaccatcaaggttgtgggcaacaatgc 1870
tgcaattggtaactggaacactggcagtgccgtctctctttctgcttcccagtat 1925
acttcttcaaatccactgtggaccggaactgtcaacttccccgctggcacggctt 1980
tgcaattcaagttcatcaatgttgctgcaaacggtgctgtcgtatgggagagaga 2035
tcctaaccgcagctttaccgttccaagatcttgccagacttctacaagtatcaac 2090
acgagctggcaggga 2105
```

Supplementary Material 3. Sequence of ORF encoding for AmyT1 from *Tetracladium* sp. Exons are indicated in grey.
