# Supplementary material for: Purification and characterization of a novel cold adapted fungal glucoamylase
Source: Microb Cell Fact. 2017 May 2;16:75. doi: 10.1186/s12934-017-0693-x (PMC5414198; doi:10.1186/s12934-017-0693-x)
Supplement: Supplementary file 4 — Additional file 4. Molecular Phylogenetic analysis by Maximum Likelihood method. A, Based on the encoding nucleotide sequences; B, Based on the translated sequences. All positions with less than 95% site coverage were eliminated, that is, fewer than 5% alignment gaps, missing data, and ambiguous bases were allowed at any position. Evolutionary analyses were conducted using MEGA7. Box, Sequences from Tetracladium sp. [file 12934_2017_693_MOESM4_ESM.pdf]

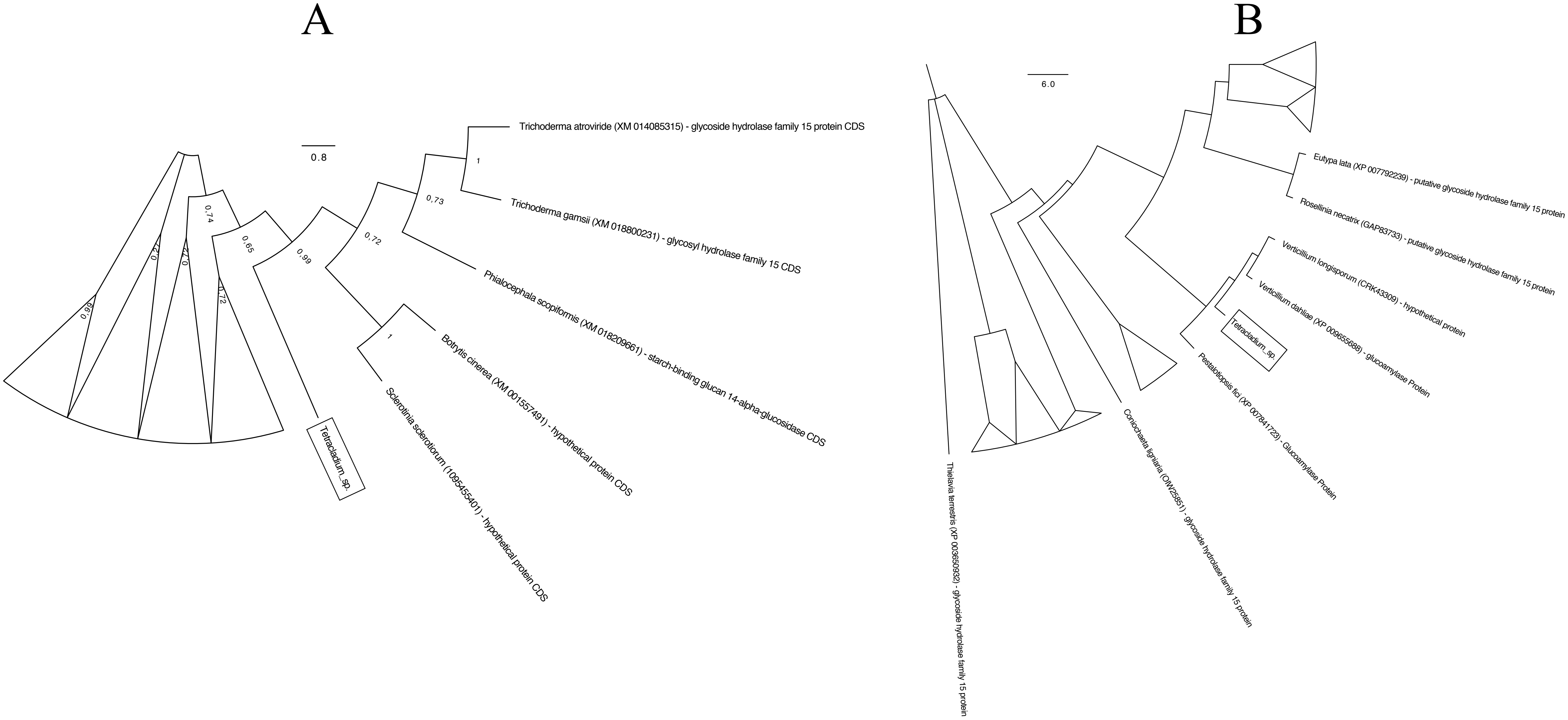

Supplementary Material 4. Molecular Phylogenetic analysis by Maximum Likelihood method. A, Based on the encoding nucleotide sequences; B, Based on the translated sequences. All positions with less than 95% site coverage were eliminated, that is, fewer than 5% alignment gaps, missing data, and ambiguous bases were allowed at any position. Evolutionary analyses were conducted using MEGA7. Box, Sequences from *Tetracladium* sp.
